# Supplementary material for: Variation in the frozen lesion size according to the non-occluded application duration and technique for cryoballoon ablation
Source: PLoS One. 2024 Jan 25;19(1):e0297263. doi: 10.1371/journal.pone.0297263 (PMC10810503; doi:10.1371/journal.pone.0297263)
Supplement: S1 File — (DOCX) [file pone.0297263.s001.docx]

**SUPPLEMENTAL MATERIALS**

| **Table of Contents** |  | Page |
| --- | --- | --- |
| **Supplemental Figure 1. cryoballlon temperature in a case with push-up technique at 20 seconds and the laminar flow during 150 seconds**  **Supplemental Figure 2. Study results according to the push-up technique**  **Supplemental Figure 3. Temperature at the time of the push-up according to the push-up timing** |  |  |
|  |  |  |
|  |  |  |
|  |  |  |
|  |  |  |
|  |  |  |
|  |  |  |
|  |  |  |
|  |  | 2 |
|  |  |  |
|  |  | 3 |
|  |  |  |
|  |  |  |
|  |  | 4 |
|  |  |  |
|  |  |  |
|  |  |  |
|  |  |  |
|  |  |  |
|  |  |  |
|  |  |  |
|  |  |  |
|  |  |  |
|  |  |  |

**Supplemental Figure 1. cryoballlon temperature in a case with push-up technique at 20 seconds and the laminar flow during 150 seconds**

**Supplemental Figure 2. Study results according to the push-up technique**

**Supplemental Figure 3. Temperature at the time of the push-up according to the push-up timing**
